# Impact of Weight Loss on Atrial Fibrillation

**DOI:** 10.7759/cureus.46232

**Published:** 2023-09-29

**Authors:** Md Ripon Ahammed, Fariha Noor Ananya

**Affiliations:** 1 Internal Medicine, Icahn School of Medicine at Mount Sinai, NYC Health and Hospitals, New York City Health + Hospital, Queens, New York, USA; 2 Internal Medicine, Dhaka Medical College and Hospital, Dhaka, BGD

**Keywords:** af, arrythmia, obesity, weight loss, atrial fibrillation

## Abstract

Atrial fibrillation (AF) is a prevalent and persistent irregular heart rhythm that is expected to dramatically increase in prevalence in the next few decades. Several established cardiovascular risk factors, including obesity, play a significant impact in developing AF. Obesity, characterized by a high body mass index (BMI), is particularly concerning as it directly elevates the risk of AF and other cardiovascular comorbidities. This review explores the complex interplay between obesity and AF, specifically highlighting the reversible nature of obesity-induced cardiac remodeling with weight loss. As we will soon discover, several insights from weight management offer promising strategies for AF prevention and management.

## Introduction and background

Atrial fibrillation (AF) is the most prevalent irregular heart rhythm among adults, and experts anticipate that its occurrence will nearly triple in the upcoming 30 years, resulting in a widespread AF outbreak. In the United States of America (USA), approximately 5.2 million people currently have AF, and this number is predicted to reach 12.1 million by the year 2030 [1]. Several established cardiovascular risk factors have been recognized as independent predictors of the development of AF including, but not limited to, male sex, aging, obesity, excessive alcohol use, valvular heart disease, ischemic heart disease, congestive heart failure, hypertension (HTN), left ventricular hypertrophy, obstructive sleep apnea (OSA), thyroid disease, and diabetes mellitus (DM) [2-3]. A direct correlation was reported between body mass index (BMI) and the likelihood of developing AF, with each one-unit rise in BMI (measured in kg/m^2^) linked to a 4.7% higher risk of AF [4]. Obesity, defined as having a BMI greater than 30, is also a risk factor for other diseases associated with AF, such as OSA, HTN, ischemic heart disease, and DM [3]. Weight loss has be shown to be beneficial for cardiovascular health by directly reducing the risk of AF and incidence of other cardiovascular events [5]. This review delves into the intricate relationship between obesity and AF, emphasizing the beneficial impact of weight loss on AF.

## Review

Effect of obesity on AF

AF is linked to fibrosis and increased volume of the atria, which can be caused by any of the risk factors previously mentioned [6]. As a risk factor for AF, obesity is the second most significant attributable risk factor after HTN. The burden of AF is expected to grow in the coming decades due to the global rise in obesity [7]. Each unit increase of BMI over 25 kg/m^2^ was linked to a significant risk (p-value < 0.0001) of developing AF. Meanwhile, individuals who were initially obese but successfully lost weight and achieved a BMI below 30 kg/m^2^ within five years experienced a decreased AF risk comparable to those who sustained a BMI below 30 kg/m^2^ over the same time frame [8]. Jones et al. analyzed 10 studies with 108,996 participants and found that a 5% rise in weight was correlated with a 13% higher occurrence of AF [9].

Increased weight is particularly associated with AF due to the electrical pathway and structural remodeling of the atria [6]. In the heart, obesity can cause increased left atrial volume, diastolic dysfunction, left ventricular hypertrophy, fibrosis, inflammatory markers (e.g., interleukin-6, C-reactive protein), and fat content. Such changes can lead to AF, particularly with sustained obesity. Furthermore, interestingly fatty infiltration of the posterior left atrium has been postulated as a possible mechanism of obesity induced AF [2]. In a sheep study, Lin et al. revealed that the presence of fat cells can directly affect the electrical properties and ion channels in the heart muscle cells of the left atrium, leading to an increased chance of developing AF [10]. Greater accumulation of fat tissue within the body can lead to a condition characterized by insufficient capillary formation, resulting in decreased oxygen supply, which subsequently triggers an inflammatory response and the release of cytokines. This inflammatory reaction can disrupt adipokine levels, calcium balance, and ion channel functionality and consequently increases atrial fibrosis and the onset of PV arrhythmias, ultimately culminating in AF.

The activation of critical signaling pathways, such as the renin-angiotensin-aldosterone system (RAAS), connective tissue growth factor, tissue growth factor beta (TGF-β), and endothelin-1, can promote the deposition of more collagen between cells. This heightened collagen accumulation may impede atrial conduction, creating an environment conducive to the re-entry and perpetuation of AF. In addition, autonomic dysfunction in individuals who are obese and suffer from concurrent obstructive sleep apnea can also be a precipitating factor for AF [8,11]. An ovine study showed cardiac structural and electrophysiological reverse remodeling with a 30% weight reduction [12]. In a human study, Munger et al. aimed to examine the electrophysiological and hemodynamic features of the pulmonary veins and left atrium in obese patients with AF. The researchers compared patients with a normal BMI to those with a BMI of 30 or higher and revealed that obese individuals exhibited a reduced effective refractory period in both the left atrium and pulmonary vein, along with elevated left atrial pressure and volume and diminished left atrial strain. These factors could potentially play a role in the onset and continuance of AF in obese patients [13]. Moreover, AF is harder to treat in individuals with larger atria because the increased atrial space provides more room for abnormal electrical patterns to develop (multiple wavelet hypothesis), making it less responsive to treatment [14].

Impact of weight loss on AF

Abed et al. conducted a randomized controlled trial of 150 symptomatic patients who were overweight and obese with AF. These patients were assigned randomly to either general lifestyle advice or weight management intervention. Over a 15-month period, they were under intensive management of cardiometabolic risk factors. The burden and severity of AF symptoms were the primary outcomes, which were assessed every three months. The results revealed that the intervention group had a significant weight loss, a decrease in the severity and burden of symptoms related to AF, and positive changes in their heart's structure. Overall, this study suggested that weight loss combined with intensive risk factor management (RFM) can effectively help manage AF [15]. Pathak et al. studied 1,415 individuals with AF to investigate the role of weight reduction and weight fluctuation on rhythm control in obese patients. They focused on 355 participants and categorized their weight loss into three groups: ≥10% vs. 3-9% vs. <3% weight loss. The results revealed that the burden of AF was significantly reduced and maintained in sinus rhythm in the group of sustained weight loss. Particularly, those in the ≥10% weight loss group had a six times higher chance of arrhythmia-free survival in comparison to the other two groups [16].

Park et al. analyzed eight studies involving 1,425 patients with AF postablation and found that weight loss might lead to a lower recurrence of AF, particularly in patients who had ≥10% weight reduction from baseline, had a shorter history of AF (less than 12 months), and had undergone weight loss before the ablation procedure [17]. A study conducted by Mohanty et al. aimed to investigate the effects of weight loss on the longstanding persistent AF (LSPAF) managed with catheter ablation (CA). The participants received a personalized diet plan and a moderate-intensity exercise program, totaling 150 minutes per week, under the guidance of a dietician. They were also required to maintain diet and exercise records and attend compliance visits. Out of 90 obese LSPAF patients, 58 volunteered for the intervention for weight loss lasting up to one year, while 32 served as the control group. Over the study period, the patients' body weight was measured every six months, and arrhythmia status was assessed using various methods. The results showed that weight loss led to a significant improvement in the patients' quality of life, but it had no impact on the severity of symptoms or the long-term outcome of the ablation procedure [18].

In a study led by Yew Ding et al., clinic patients with specialized arrhythmia were divided into two groups: one following the "intermittent fasting 5:2 diet" (diet group) and a control group receiving no specific dietary advice. The progress of both groups was monitored through phone calls and patient diaries. The diet group achieved significant weight loss prior to AF ablation, and after one year, both groups had similar rates of AF recurrence. This study suggests that significant weight reduction can be achieved in obese AF patients within a specialized arrhythmia clinic setting, even with unsupervised dietary advice. Furthermore, it highlights that medium-term success rates were excellent, and procedural complications were rare [19]. The SORT-AF study involved a medical weight loss program that included nutritional guidance and exercise training, along with patient diary maintenance and compliance visits. It investigated the impact of weight reduction on AF ablation outcomes. The study involved 133 patients with symptomatic AF who underwent AF ablation and having a BMI between 30 and 40 kg/m^2^, were randomized to either usual care or weight reduction. These findings indicated that a decrease in weight resulted in a notable decrease AF burden following ablation. Moreover, a substantial association was observed between BMI and AF recurrence among patients with persistent AF in contrast to those with paroxysmal AF. The study highlights the importance of lifestyle management as a supplement to AF ablation in obese patients with persistent AF [20]. 

Abed et al. performed a randomized controlled trial involving overweight and obese outpatient individuals experiencing symptomatic AF. Their aim was to investigate how reducing weight and addressing factors for cardiometabolic affect AF burden and cardiac structure. This study involved subjects with a strict low-calorie diet, where meal replacements were provided, which were gradually replaced with low-glycemic index foods. Along with this, a written exercise plan was introduced and increased over time. Patients recorded their progress in a diary and attended additional appointments [16]. The summary of the studies included in our review illustrating the impact of weight loss on AF is shown in Table 1.

**Table 1:** List of studies illustrating the effect of weight loss on AF AF: atrial fibrillation

Study author	Study type	Participants (n)	Intervention/comparison	Outcomes
Abed et al. [15]	Randomized controlled trial	150 symptomatic AF patients	Intervention: Weight management program involving exercise, low-calorie diet, and cardiometabolic risk factor management. Comparison: General lifestyle advice.	1. Decreased severity and burden of atrial fibrillation (AF) symptoms. 2. Positive changes in cardiac structure noted.
Pathak et al. [16]	Observational study	355 patients with AF	Three groups based on weight loss: ≥10%, 3-9%, <3% weight loss	1. Sustained weight loss (≥10%) associated with significantly lower AF burden. 2. Higher arrhythmia-free survival in the ≥10% weight loss group.
Park et al. [17]	Meta-analysis	1,425 AF patients post-ablation	Analyzed weight loss impact on AF recurrence based on different factors	1. Weight loss, especially ≥10% of the initial weight, linked to lower recurrence of AF. 2. Particularly effective in patients with shorter AF history (<12 months) and weight loss before ablation.
Mohanty et al. [18]	Interventional study	90 obese longstanding persistent AF (LSPAF) patients	Weight loss intervention with personalized diet and moderate-intensity exercise. Control group declined intervention.	Losing weight enhanced the quality of life for patients, yet it did not have any influence on the severity of symptoms or the ultimate success of long-term ablation.
Yew Ding et al. [19]	Observational study	Specialized arrhythmia clinic patients	Diet group (intermittent fasting 5:2 diet) and control group receiving no dietary advice.	1. Significant weight loss achieved in the diet group. 2. Similar AF recurrence rates in both groups after one year.
Gessler et al. [20]	Randomized controlled trial	133 symptomatic AF patients with BMI 30-40 kg/m²	Intervention: Medical weight loss program including nutritional guidance, exercise training, patient diary, and compliance visits. Comparison: Usual care.	1. Decrease in weight led to a substantial reduction in both BMI and AF burden following ablation. 2. BMI had a substantial association with AF recurrence in the patients with persistent AF.

Impact of different weight reduction strategies

In an observational study including 355 patients with symptomatic AF and a BMI of ≥27 kg/m^2^, Pathak et al. implemented physician-directed RFM and arrhythmia follow-up. The results indicated that the RFM group exhibited higher arrhythmia-free survival, fewer hospitalizations, reduced drug use, improved quality of life, and cost savings [21]. In a similar vein, Pathak et al. (2014) conducted an original article involving 61 patients undergoing AF ablation with a BMI of ≥27 kg/m^2^ and at least one cardiac risk factor. Physician-directed RFM was implemented according to guidelines. The study found that the RFM group experienced significant improvements, including weight loss, improved blood pressure, improved glycemic control, reduced AF duration, frequency, symptoms, and better arrhythmia-free survival [22]. Moving beyond traditional RFM, Jamaly et al. (2016) conducted a multicenter case-control study involving 4,021 obese people with sinus rhythm and no AF history. The findings indicated that patients who had bariatric surgery were found to have 29% lower risk of first-time AF diagnosis compared to the control group [23]. In a more recent prospective cohort study, Donnellan et al. (2020) investigated the impact of bariatric surgery on AF in 220 morbidly obese patients (BMI ≥ 40 kg/m^2^). The study revealed a remarkable reversal of the AF type in 71% of gastric bypass patients, 56% of sleeve gastrectomy patients, and 50% of gastric banding patients [24]. The studies illustrating the impact of different weight reduction strategies are summarized in Table 2.

**Table 2:** List of studies demonstrating the impact of different weight reduction strategies on AF AF: atrial fibrillation

Author	Type of study	Study population	Intervention	Interpretation
Pathak et al. [21]	Observational study	355 patients with symptomatic AF and BMI ≥ 27 kg/m^2^	Physician-directed risk factor management (RFM) and arrhythmia follow-up	The RFM group had higher arrhythmia-free survival, fewer hospitalizations, reduced drug use, improved quality of life, and cost savings.
Pathak et al. [22]	Original article	61 patients undergoing AF ablation with BMI ≥ 27 kg/m^2^ and ≥1 cardiac risk factor	Physician-directed RFM according to guidelines	The RFM group had weight loss, improved blood pressure, better glycemic control, reduced AF duration, frequency, symptoms, and better arrhythmia-free survival.
Jamaly et al. [23]	Multicenter case-control	4,021 obese patients with sinus rhythm without history of AF	Bariatric surgery	Patients who had a bariatric surgery had a 29% lower risk of first-time AF diagnosis compared to the control group.
Donnellan et al. [24]	Prospective cohort study	220 morbidly obese individuals (BMI ≥ 40 kg/m^2^) before and after bariatric surgery	Bariatric surgery	AF-type reversal was found to occur in 71% of gastric bypass patients, 56% of sleeve gastrectomy patients, and 50% of gastric banding patients. Cox proportional hazards analysis showed a major association between percentage weight loss and AF-type reversal.

Collectively, these studies underscore the importance of addressing obesity as a risk factor in AF management. The findings highlight the effectiveness of RFM alongside bariatric surgery in improving AF outcomes, including symptom reduction, glycemic control, and AF-type reversal. These insights are pivotal in guiding clinical interventions and preventive strategies for AF, particularly in individuals with obesity-related risk factors.

## Conclusions

Obesity is an ongoing pandemic, and it is a pivotal risk factor in the burgeoning AF epidemic. The escalating prevalence of AF is inextricably linked to the global obesity crisis. Obesity induces structural and electrophysiological changes within the heart, culminating in AF. However, there is a ray of hope in reversing of these changes through weight loss interventions. Studies demonstrate substantial reductions in AF burden and symptom severity post-weight loss. These findings emphasize the urgency of addressing obesity as a cornerstone in AF prevention and management. Proactive measures, including weight management and surgical interventions, hold the promise of mitigating AF's impact on global cardiovascular health.
